# Robustness of birth-death and gain models for inferring evolutionary events

**DOI:** 10.1186/1471-2164-15-S6-S9

**Published:** 2014-10-17

**Authors:** Maureen Stolzer, Larry Wasserman, Dannie Durand

**Affiliations:** 1 Department of Biological Sciences, Carnegie Mellon University, Forbes Ave, Pittsburgh, PA, 15213, USA; 2 Department of Statistics, Carnegie Mellon University, Forbes Ave, Pittsburgh, PA, 15213, USA; 3 Department of Computer Science, Carnegie Mellon University, Forbes Ave, Pittsburgh, PA, 15213, USA

**Keywords:** birth-death, ancestral state reconstruction, bootstrapping, domains

## Abstract

**Background:**

Phylogenetic birth-death models are opening a new window on the processes of genome evolution in studies of the evolution of gene and protein families, protein-protein interaction networks, microRNAs, and copy number variation. Given a species tree and a set of genomic characters in present-day species, the birth-death approach estimates the most likely rates required to explain the observed data and returns the expected ancestral character states and the history of character state changes. Achieving a balance between model complexity and generalizability is a fundamental challenge in the application of birth-death models. While more parameters promise greater accuracy and more biologically realistic models, increasing model complexity can lead to overfitting and a heavy computational cost.

**Results:**

Here we present a systematic, empirical investigation of these tradeoffs, using protein domain families in six metazoan genomes as a case study. We compared models of increasing complexity, implemented in the Count program, with respect to model fit, robustness, and stability. In addition, we used a bootstrapping procedure to assess estimator variability. The results show that the most complex model, which allows for both branch-specific and family-specific rate variation, achieves the best fit, without overfitting. Variance remains low with increasing complexity, except for family-specific loss rates. This variance is reduced when the number of discrete rate categories is increased.

Model choice is of greatest concern when different models lead to fundamentally different outcomes. To investigate the extent to which model choice influences biological interpretation, ancestral states and expected events were inferred under each model. Disturbingly, the different models not only resulted in quantitatively different histories, but predicted qualitatively different patterns of domain family turnover and genome expansion and reduction.

**Conclusions:**

The work presented here evaluates model choice for genomic birth-death models in a systematic way and presents the first use of bootstrapping to assess estimator variance in birth-death models. We find that a model incorporating both lineage and family rate variation yields more accurate estimators without sacrificing generality. Our results indicate that model choice can lead to fundamentally different evolutionary conclusions, emphasizing the importance of more biologically realistic and complex models.

## Background

Analysis of genomic characters in the context of a species phylogeny is a rich source of insight into genome evolution. Parsimony methods for ancestral state reconstruction are well established, but can lead to incorrect conclusions when the data does not satisfy the underlying assumptions and cannot be used for inferring rates of evolution. Probabilistic approaches for ordered, discrete characters have been adapted from birth-death Markov models of population size [1] and have been used to model the evolution of gene, protein, fold, and domain family sizes [2], protein-protein interaction networks [3,4], microRNAs [5] and copy number variation [6]. Probabilistic models of genome character evolution have been further elaborated by integrating birth-death models in a phylogenetic context [7-14,6].

Developing such models requires achieving a balance between conflicting goals: A model must capture the important features of the biological system. The calculations required to estimate the associated probabilities must be theoretically feasible. From a practical perspective, it must be possible to estimate the parameters in a reasonable amount of time without overfitting. Mathematical features added for computational convenience may have unintended biological consequences and these must be avoided.

Current phylogenetic birth-death models differ in how they approach these trade-offs. An implicit assumption in the birth-death framework is that all families were present in the common ancestor. This assumption is avoided in models that also include a *gain *or *innovation *event, which allows for later emergence of new families and can also be used to model horizontal transfer. To model family evolution in genomes at equilibrium, Hahn and colleagues [15,16] added the assumption that the birth and death rates are the same. The resulting model can be used to identify families that reject the hypothesis of neutral evolution, but is not well suited to genomes undergoing rapid expansion (e.g., whole genome duplication) or contraction (e.g., genome collapse in emerging pathogens). Evidence for both lineage-specific and family-specific rate variation for many processes of genome evolution, including substitution rates [17], genomic rearrangements [18], and gene duplication [16], suggests that rate variation is an important property to model. Some models require that a tree with branch lengths be provided; others infer branch lengths from the data. Our current understanding of these trade-offs on real data sets is limited.

Here, we compare the robustness and stability of three models of increasing complexity using Count [8], software that offers one of the most general phylogenetic birth-death and gain models currently available. Count's event model captures gains of novel families, as well as expansion and contraction of existing families. It accommodates rate variation across phylogenetic lineages and across families, does not assume equality of birth and death rates, and does not require an ultrametric tree with known branch lengths.

We investigated the impact of model complexity on model fit and the stability of the estimators using a benchmark data set of 4650 PFAM families in six high-quality, well-annotated metazoan genomes (provided in Additional file 1). We compared the importance of branch-specific and family-specific rate heterogeneity to the stability of the model, and assessed the impact of increasing the number of rate categories on estimator variance. We also considered to what extent a more complex model could lead to fundamentally different evolutionary conclusions.

## Methods

### Birth-death-gain model

In this study, we used the Count software package [8] to investigate the impact of model complexity on robustness and stability. Count takes as input a species tree, *T *= (*V, B*), with nodes *V *and branches *B*, and a set of phylogenetic profiles representing each family on *L*(*T*), the leaves of *T *. Each phylogenetic profile is a vector of length |*L*(*T*)|, such that element *s *of the profile corresponds to the number of members of that family in species *s *∈ *L*(*T*).

The Count analysis proceeds in two passes. In the first pass, Count estimates the parameters of the model by likelihood maximization. The parameters include the event rates, the lengths of the branches in *T*, if these are not specified by the user, and the distribution of ancestral family sizes at the root of *T*. In all models, family sizes on the root are assumed to follow a Poisson distribution with mean Φ. In the second pass, Count uses the estimated parameter values to calculate the expected size of each family in every ancestral species (i.e., every internal node of *T*) and the expected number of gain, loss, expansion, and contraction events along each branch in *B*.

Count's birth-death and gain model is a continuous-time Markov process, with a transition probability that depends on the branch length, *t*, and the rates of Expansion (λ), Gain (κ), and Loss (*µ*). A population of size *i *increases with probability (λ*i *+ κ)*t *and decreases with probability *µit*. We denote the ensemble of these model parameters by Π = {*t*, λ, κ, *µ*}.

Count offers a series of nested models of increasing complexity. In the full model, each parameter is the product of a branch component and a family component: Π = {*t_b_t_f_*, λ*_b_*λ*_f_, µ_b_µ_f_*, κ*_b_*κ*_f_*}. The number of branch parameters can be reduced by one through normalization. Count assumes unit loss rates (*µ_b _*= 1) by default. Alternatively, one may assume *t_b _*= 1 and allow the loss rates to vary, as we did in this study, resulting in Π*_b _*= {*t_b _*= 1, λ*_b_, µ_b_*, κ*_b_*}. The variation of each family-specific parameter in Π*_f _*= {*t_f_*, λ*_f_, µ_f_*, κ*_f_*} is modeled by a discretized gamma distribution with *c *rate categories, where the rate associated with each category is the mean of the corresponding quantile.

Count also allows for a model with fewer parameters under the assumption that all families evolve at the same rate within any given branch (Π*_f _*= {1, 1, 1, 1}). In the simplest model, each parameter takes on a single value for all families and all branches: Π^* ^= {1, λ^*^, µ^*^, κ^*^}.

### Data

We use protein domain families as test data for this study. Domains are sequence fragments that encode protein modules with a distinct structure and function, the basic building blocks of proteins. Here, we use the term "domain" as an abstraction of a particular structural fold or functional motif and define a domain family to be the set of all instances of that domain in a given set of proteomes. The set of all domains encoded in a genome can be viewed as the protein function toolkit of the species. For this study, we are not concerned with how domains are distributed across individual proteins. The history of the domain family complement provides a view of the evolution of the functional capabilities of the proteome.

**Figure 1:**
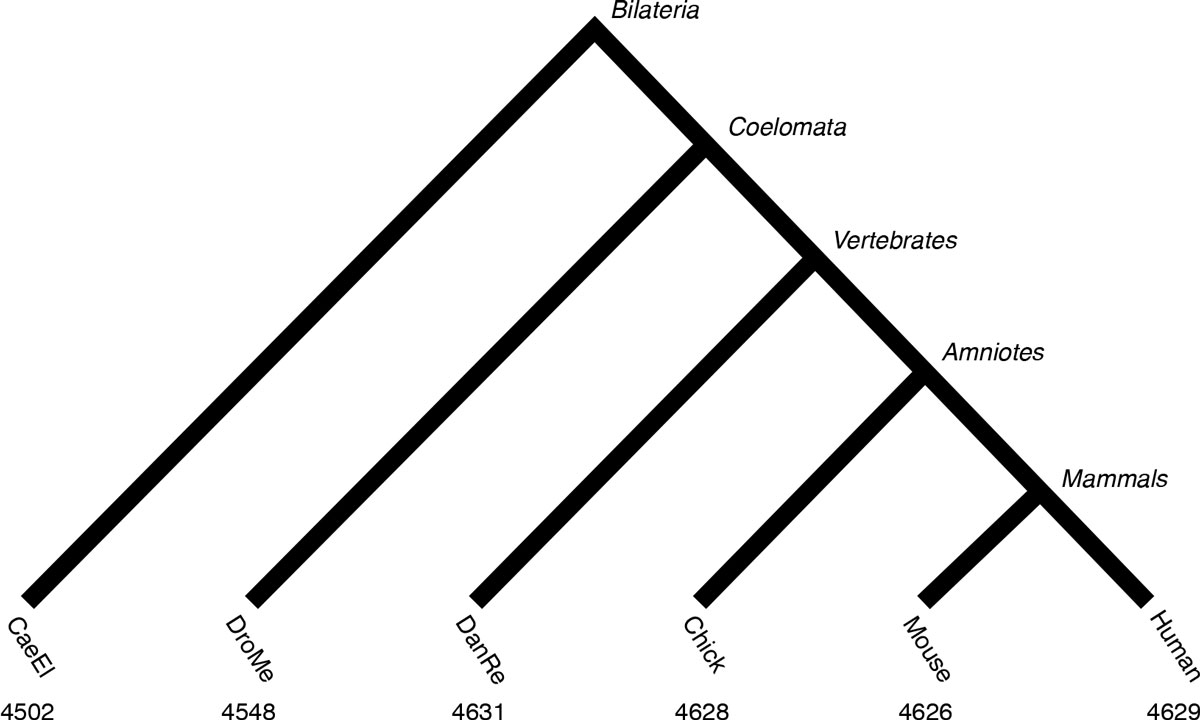
**Species tree and family sizes.** Species tree for the six genomes analyzed in this study, annotated with the number of protein domain families in each genome.

In this case study, we considered the evolution of domain family sizes in six completely sequenced genomes from two invertebrate (worm and fly) and four vertebrate species (human, mouse, chicken, and zebrafish). We chose these species because of the size and the complexity of domain families encoded in vertebrate genomes. Further, these are well-studied genomes with good annotation quality, reducing the risk that annotation errors will confound the analysis. The species tree for these genomes (Figure 1) reflects the coelomate hypothesis supported by Zheng et al. [19]. This branching order is controversial; increasing evidence supports the ecdysozoa hypothesis placing nematodes and flies as sister groups [20]. Since we focus here on model fit, robustness, and inference rather than biological interpretation, the structure of the tree is less of a concern.

For the purpose of this study, we defined domain families in terms the PFam database [21]. The genomic sequences for *Homo sapiens *(Human), *Mus musculus *(Mouse), *Gallus gallus *(Chick), *Danio rerio *(DanRe), *Drosophila melanogaster *(DroMe), and *Caenorhabditis elegans *(CaeEl) were downloaded from the Panther 7.0 database [22]. Domain families in these six species were identified by scanning the genomic sequences with the set of identifying HMMs from the PFam 24.0 database [23], using the default settings. The size of a domain family in a given species is defined to be the number of amino acid sequence fragments annotated with a given PFam family identifier. This resulted in phylogenetic profiles of 4,650 PFam families that appear in at least one of the six genomes used in the study (see Additional file 1). Of these, 2476 families (53%) are present in all six species and 336 families (7%) are unique to a single species. Mean family sizes ranged from 3.7 in fly to 8.6 in mouse. While most domain families are relatively small (less than 15 copies per species), a few are very large (see Figure S1 in Additional file 2). For example, the zinc fingers and the WD40 domain family have 6,799 and 5,192 members, respectively. This ensemble of phylogenetic profiles, together with the species tree (Figure 1), formed the input of our birth-death analyses.

### Inference

We inferred the rates of domain family expansion, gain, and loss for four models of increasing complexity - the Constant model, the Lineage model, and the Family-Lineage model with two rate categories (FL2) and with three rate categories (FL3) - defined as follows:

**Constant (C) model**: Rates are constant across lineages and families; Π = Π^* ^in all species and for all families. This model has four parameters: λ^*^, µ^*^, κ^*^, and Φ. Recall, that t^* ^= 1.

**Lineage (L) model**: Rates vary across lineages, but are constant across families. For all families, Π = Π*_b_*, ∀*b *∈ *B*. This model has 3|*B*| + 1 parameters.

**Family-Lineage (FL) model**: Rates vary across both lineages and families. Each parameter has a branch-specific component, Π*_b_*, and a family-specific component, Π*_f_*. This model has 4*c *+ 3|*B*| + 1 parameters, where *c *is the number of rate categories.

To facilitate convergence, we carried out the parameter estimation procedure in stages of increasing model complexity, as recommended in the Count manual [24]. Initially, Π^* ^and Φ are estimated in the C model. In the second stage, the branch-specific parameters, Π*_b_*, were estimated under the assumptions of the L model, using the values of Π^* ^from stage one as initial estimates. In the third stage, all parameters were (re)estimated in the FL2 model, using the estimates from the previous stage (the L model) as a starting point. Finally, the parameters were estimated using the FL3 model, with initial estimates obtained from the FL2 parameter values. At each stage, parameters were estimated using an iterative, numerical optimization procedure [25] that terminates when the increase in ln(*L*) between consecutive iterations is less than 0.01. Following each estimation stage, the expected events and family sizes were calculated from the posterior probabilities.

To assess model fit, we calculated the Bayesian Information Criterion [26], BIC = *m *ln(*n*) − 2 ln(*L*), and the Akaike Information Criterion [27], AIC = 2*m *− 2 ln(*L*), where *m *is the number of parameters and *n *is the number of domain families. We used the bootstrap [28] to evaluate the variance of the estimators. Each bootstrap replicate was constructed by sampling 4,650 phylogenetic profiles with replacement. We generated 100 bootstrap replicates and inferred event rates for each replicate, following the full four-stage estimation procedure described above. For one bootstrap replicate, the gain and loss rate estimators were more than nine standard deviations from the mean on almost all branches in the L model. Therefore, we removed this outlier from further analysis. The expected ancestral states and events were calculated using the estimated parameters for each of the remaining 99 replicates. From the resulting distributions, the variance and standard error were calculated for all inferred parameters, events, and ancestral nodes. Count returned "NaN" for 58 domain families (1.2%) during the second pass for the FL3 model. These families were removed from all models when comparing the results from the second pass.

The recommended progression in increasing model complexity is to introduce first lineage-specific rate variation and then add family-specific rate variation. For comparison, we also introduced a model that incorporates only family-specific rate variation. Under the Family Only (**FO**) model, rates vary across families, but not lineages. Each parameter has a family-specific component, Π*_f_*, and a branch component, Π*_b _*= Π^*^, that is constant across all species: Π = Π*_f_*Π^*^. This model has 4*c *+ 4 parameters. Parameters were estimated for the FO model with two rate categories (FO2) using Π^* ^from the C model as an initial estimate.

## Results and discussion

### Robustness and model complexity

For birth-death models in general, and Count in particular, model choice involves balancing a more descriptive model and more accurate parameter estimation against increased running time and the risk of overfitting. To better understand the nature of this trade-off for our benchmark, we first considered whether the more complex models improved fit without sacrificing generalizability, as assessed by ln(*L*), AIC, and BIC.

**Figure 2:**
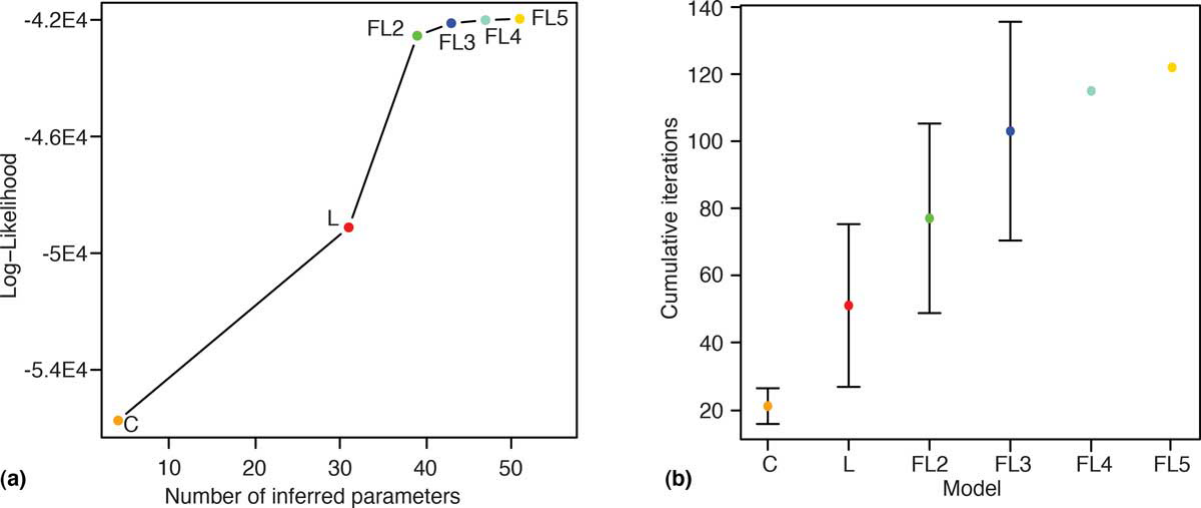
**Parameter estimation and convergence.** (a) Log-likelihood values as a function of the number of inferred parameters for the C (orange), L (red), FL2 (green), FL3 (blue), FL4 (teal), and FL5 (yellow) models. (b) The total number of iterations required to reach convergence for each model, including iterations in all previous stages. Error bars represent standard errors calculated from the bootstrap replicates.

According to all three measures (Figure 2 and Table S1 in Additional file 2), models incorporating both family- and lineage-specific rate variation represent a dramatic performance improvement over models that incorporate only one of these. Increasing the number of rate categories from two to three results in an additional performance increase, but this improvement is relatively modest. To determine whether further increases in the number of rate categories yield additional benefits, we also tested models with four (FL4) and five (FL5) rate categories. As seen in Figure 2, increasing the number of rate categories above three does not appreciably improve model fit.

The time required to complete each model stage depends on the number of iterations required to reach convergence and the time per iteration. In our study, Count's numerical likelihood maximization procedure converged for all models for the original data set and for all bootstrap replicates. The total number of iterations increased with model complexity (Figure 2 (b)). In the transition from the C to the L model, a substantial increase in the number of iterations was offset by a small increase in the time per iteration, so that the overall increase in running time was relatively small. The number of additional iterations required by the Family-Lineage models is modest, increasing by a factor of 1.5, on average, for each additional rate category. Fitting family rates in each iteration took much longer. The mean time per iteration was 34 minutes for FL2 and 57 minutes for FL3, resulting in a doubling of total running time for each additional rate category.

The number of iterations required to reach convergence varies considerably across boot-strap replicates. Interestingly, bootstrap replicates that required a large number of iterations to reach convergence in one stage converged quite quickly in the next stage, and vice versa (Figure S3 in Additional file 2). A possible explanation for this is that spending more time in one stage results in a better parameter estimate, leading to faster convergence in the next stage.

**Figure 3:**
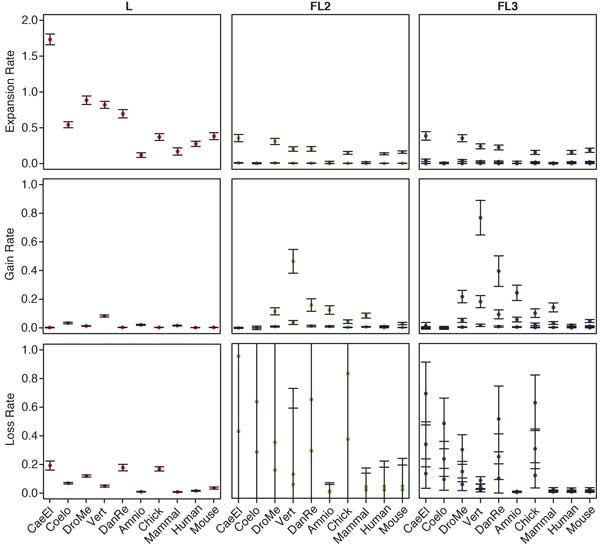
**L, FL2, and FL3 model event rates.** Event rates (colored dots) estimated with the L, FL2, and FL3 models. For the Family-Lineage models, the mean rate for each rate category is shown. Error bars represent standard errors calculated from the bootstrap replicates.

In general, increasing the number of model parameters will result in a better fit to the data, but may also increase the standard error of the estimators. Scanning Figure 3 from left to right shows the change in standard error with increasing model complexity. For Expansions, the standard error is modest on all branches and for all models. For Gains, the standard error in both Family-Lineage models is noticeably higher than in the Lineage model, especially in the highest rate categories. The standard errors associated with the lowest rate categories remain low, however. This is possibly the result of the long tails in the gamma distributions for Π*_f_*, where the fastest rate categories cover a wider range of rates than the lower categories, which are more densely clustered near zero. In contrast, the standard errors associated with the Loss rate estimator are extremely large in FL2.

**Figure 4:**
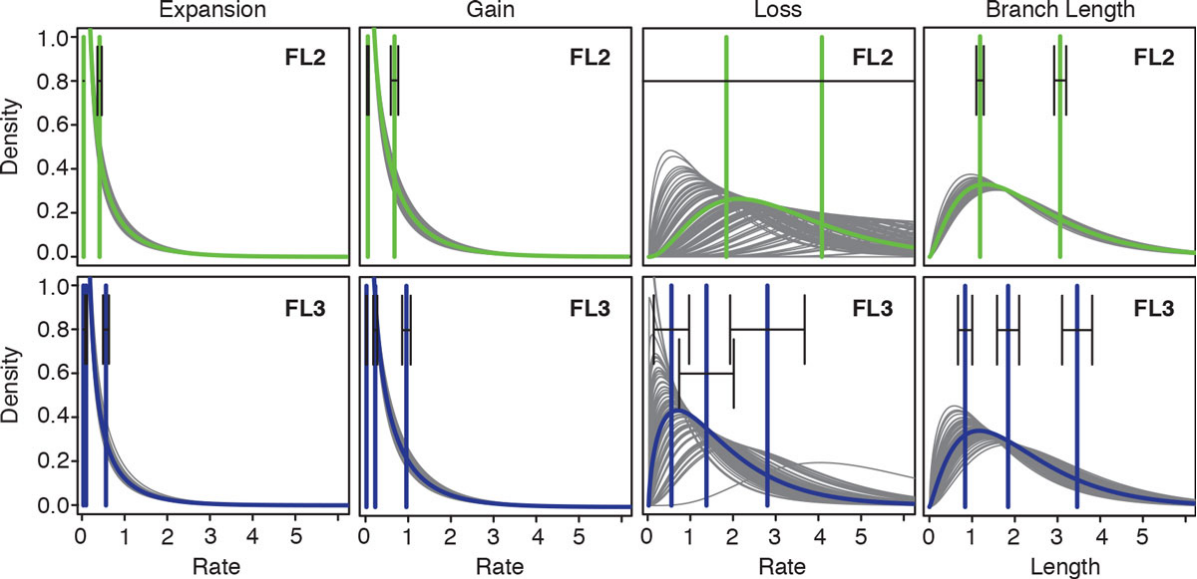
**Gamma probability distributions for family-specific parameter estimates**. Estimated distributions for the family components of the Expansion rate, Gain rate, Loss rate, and Branch length parameters. Distribution for the original data shown in green (blue) for the FL2 (FL3) model. Distributions for all bootstrap replicates shown in gray. Green (blue) vertical lines represent the mean value of the estimator for each category, based on the original data for the FL2 (FL3) model. Error bars represent standard errors calculated from the bootstrap replicates.

To better understand estimator variance, we plotted the gamma distributions specified by the shape parameter estimators inferred for each bootstrap replicate under the FL2 and FL3 models (Figure 4). For the Gain and Expansion rates, the gamma distributions are quite similar and the standard errors of the means of all rate categories are small. Interestingly, there is little difference between the means of the two lowest rate categories in the FL3 model. The Branch length distributions are more variable, especially in the FL3 model, but the standard errors are still fairly low. In contrast, the Loss rate distributions are extremely variable in shape. For the FL2 model, the standard errors are so large that they exceed the boundaries of the figure. Adding a third rate category substantially reduces, but does not eliminate, this variance. The good news is that despite large standard errors in the inferred rates, the estimates of the expected events varied much less (Figures S11-S16 in Additional file 2). Moreover, the expected events inferred with the L and FL models, although quantitatively different, had similar trends.

Comparing the FL2 and FL3 models suggest that two rate categories are adequate to model the Expansion and Gain rates; a third category confers little additional benefit for these events. In contrast, two categories are insufficient to model Loss rates, and Loss rate variance is still high in the FL3 model. Inspired by these observations, we also tested a model with two rate categories for Expansions, Gains, and Branch lengths, and four rate categories (FL2242) for Losses. Surprisingly, this model did not lead to greater stability. Increasing from two to four rate categories does not reduce the standard errors of the Loss rates (Figures S7 and S8 in Additional file 2). Moreover, these standard errors are substantially larger in the FL2242 model than in the FL3 model. Note that while the standard errors of the Loss rates decreased when the number of rate categories increased from two to three, the standard error of the family-specific Branch lengths increased. Since the probability of an event occurring on a branch depends on both the event rate and the family-specific Branch length, *t_f _*, it is possible that including a third Branch length category facilitates Loss rate inference, and that this flexibility is lacking in the FL2242 model.

We also compared the Family Only model with the Lineage and Family-Lineage models. Interestingly, the FO model obtained substantially better log-likelihood and BIC scores than the L model (Figure S2 in Additional file 2). Despite good performance with respect to general measures, a more careful look at the behavior of the FO2 model reveals poor convergence properties. In the first pass, the standard errors for the Expansion and Loss rates (Figures S5 and S6 in Additional file 2) are high compared with all other models, which is particularly striking given the small number of parameters associated with this model. In the second pass, Count was unable to make rate category assignments for 195 families (4.5%) with the FO2 model, returning "NaN" for these families. Further, while the expected gains and losses obtained with the L, FL2, and FL3 models exhibited similar trends, the FO2 model yielded a very different pattern of expected events (Figures S11-S16 in Additional file 2). For example, the FO2 model generally predicted much higher levels of gene family Gain and Expansion. In summary, the variation in convergence times and the high standard errors in inferred rates suggest that the FO2 model lacks stability, consistent with a weakly defined, multimodal likelihood landscape. This poor performance is not surprising, given the wealth of evidence for lineage-specific rate vatiation in the published literature.

### Impact of model choice on biological interpretation

Using a more complex model results in a better fit, but does it lead to fundamentally different conclusions or to similar conclusions that differ only in degree? To explore the extent to which biological conclusions are influenced by model choice, we compared the expected ancestral domain content and the expected events predicted by the C, L, FL2, and FL3 models (Figures S11-S16 in Additional file 2). Note that the goal of the following discussion is to examine the extent to which model choice could influence the conclusions of genome evolution studies. It is not our intent in this paper to make any definitive statements about domain family evolution in bilateria.

**Figure 5:**
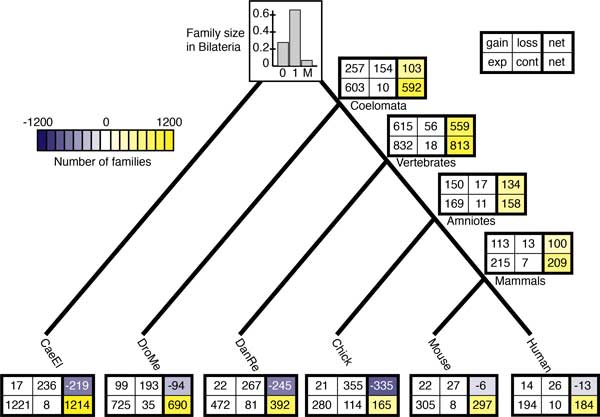
**Expected events along each branch under the L model**. The expected number of families gained, lost, expanded (exp), and contracted (cont), as well as the net change. The expected fraction of families with 0, 1, or More than one (M) domain in the bilaterian ancestor is shown on the root.

**Figure 6:**
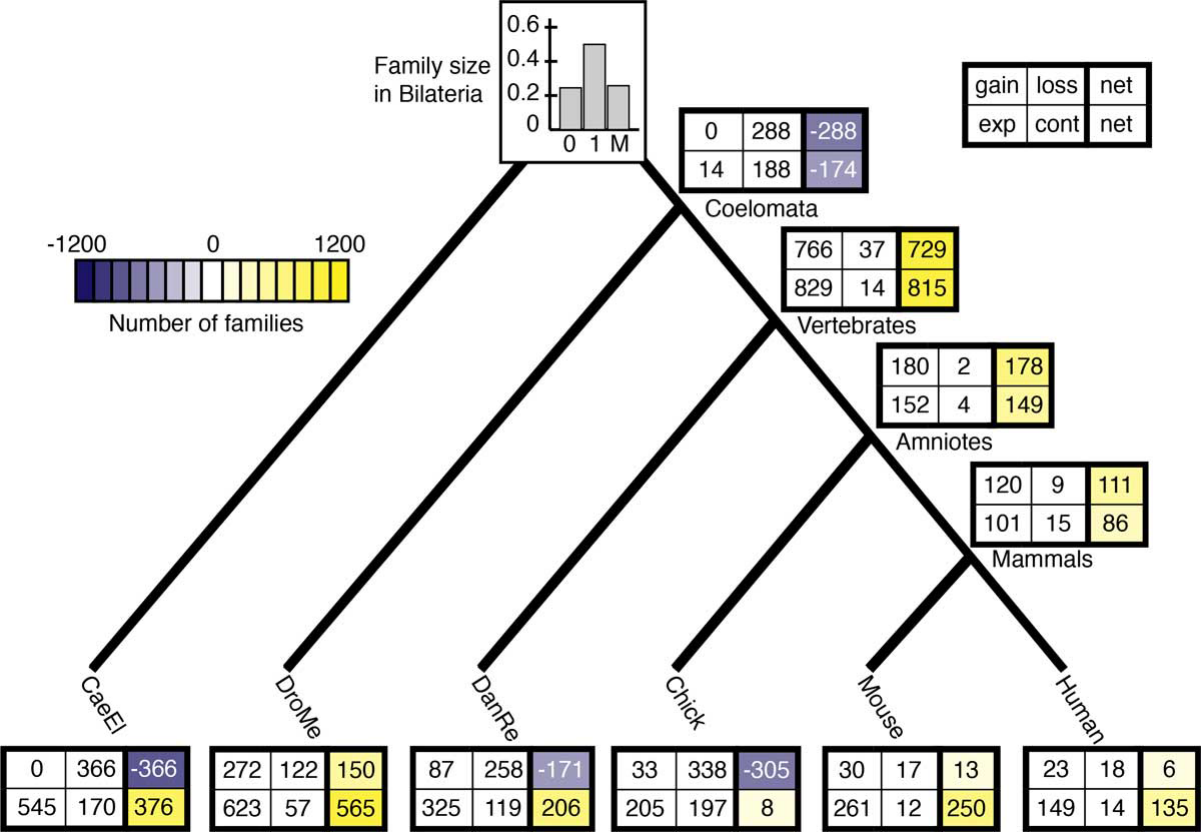
**Expected events along each branch under the FL2 model**. The expected number of families gained, lost, expanded (exp), and contracted (cont), as well as the net change. The expected fraction of families with 0, 1, or More than one (M) domain in the bilaterian ancestor is shown on the root.

Within the amniotes, all four models predict similar patterns of domain family Gain, Loss, Expansion and Contraction (Figures 5 and 6 and Figures S9 and S10 in Additional file 2), although there are quantitative differences. All models predict a net Gain of families on the branches leading to amniotes and mammals, a net Loss in the fish and chicken lineages, and little change in mouse and human. The expected number of families that expanded and contracted on these branches are also similar across the L, FL2, and FL3 models.

In contrast, a comparison of Figures 5, 6, and S10 (Additional file 2) reveals that the evolutionary trends associated with the basal lineages are qualitatively different. In the Lineage model (Figure 5), the dominant trend is ancestral genome expansion, with a net Gain of 100 families and Expansion of 600 families in the coelomate lineage and an even stronger pattern of Gain and Expansion in the vertebrate ancestor. In the invertebrate lineages, net family Loss combined with family Expansion is predicted. This pattern is especially pronounced in worm, with more than 200 Losses and less than 20 Gains, on the one hand, and a whopping 1200 Expansions on the other. In the Family-Lineage models (Figure 6 and Figure S10 in Additional file 2), Loss and Contraction dominate in the coelomate lineage, instead of a pattern of genomic elaboration. No families were gained and very few grew in size. On the branch leading to vertebrates, the number of net expected Gains is 30% larger than in the Lineage model. The worm lineage is characterized by Loss, as before, but to a much greater extent. In fly, on the other hand, the trend is reversed: The expected net change is positive, with a net Gain of ~150 families.

Overall, the evolutionary trends predicted by the two models are quite different. In the Lineage model, the protein domain complement expanded in the lineages leading to the coelomate and vertebrate ancestors. The pattern in the invertebrates is consistent with specialization, with a reduction in the total number of families and Expansion in the size of families that were retained. The Family-Lineage models suggest Contraction on the coelomate lineage and re-expansion on the branch leading to vertebrates. More Losses and Contractions and fewer Expansions are predicted in the worm genome, a pattern that is more suggestive of reduction than specialization. The net expected Gains in the fly genome, on the other hand, indicate an Expansion of the protein toolkit in that lineage. The Family-Lineage model also suggests different genome dynamics: the reduction in domain families in coelomates, followed by Expansion in the vertebrate and fly lineages, implies ongoing domain family turnover during protein evolution, compared with the Lineage model which predicts steady expansion.

In summary, for our data set, adding family-specific rate variation to the model suggests qualitatively different conclusions about genome evolution, at least for some lineages. In contrast, comparison of the FL2 and FL3 models shows that adding a third rate category does not change the interpretation of the data in a fundamental way.

## Conclusions

The recent development of phylogenetic birth-death models represents an important advance for studying the evolution of gene families and other census-type characters on a genome scale. Models have been proposed that vary in the genomic properties modeled, the number of parameters that must be estimated, and the simplifying assumptions used to make parameter inference tractable. A better understanding of how these compromises influence outcomes is important for guiding future method development and genomic analyses. Here we report an empirical case study of the impact of model complexity on model fit and the variability of the estimators. Using Count [8], a program that implements one of the most general birth-death models available, we investigated the influence of branch- and family-specific rate variation on outcomes in a typical genomic data set. Model fit was evaluated with respect to the likelihood, AIC, and BIC. In addition, we used a bootstrapping approach to assess the variability of the estimators.

This is, to our knowledge, the most comprehensive evaluation of model choice for genomic birth-death models to date and the first to assess estimator variance in a systematic way. Several authors have commented on aspects of model choice and robustness observed in the course of a particular biological analysis [25,29]. A number of articles announcing new birth-death models or software include empirical studies comparing the behavior of the new software with existing software [12,14,6]. However, none of these studies represent a comprehensive characterization of the impact of model choice on the robustness of birth-death models.

Our results show that a model that captures both lineage-specific and family-specific rate variation is superior, yielding more accurate estimators without sacrificing generality. Branch-specific rates alone were not sufficient to capture the rate variation in our data set. Adding a third family rate category further improved model fit, but not dramatically. The benefit of additional rate categories was negligible. Family-specific rate variation substantially improves model fit, but at a computational cost. In our study, each additional rate category roughly doubled the running time. That being said, our results suggest that a large number of rate categories are not needed. For our data set, three rate categories were sufficient.

Our bootstrapping approach reveals that the variability of the estimators increased with model complexity, as expected. Loss rates had particularly high standard errors. Estimating the parameters of birth-death Markov models using likelihood maximization requires summing over many latent variables. Latent variable models are frequently characterized by poorly defined, multimodal likelihood functions, and this appears to be the case here.

Our case study demonstrates that model selection can substantially impact model fit, estimator variance, running times and, most important, biological conclusions. While it is not clear to what extent these results can be generalized to other data sets, it certainly suggests that expanded studies on the complexity of birth-death models is a valuable direction for future work. One important course of development would be to simulate data sets with various properties and determine how well those properties can be recovered by models of increasing complexity. Tests using simulated data will allow comparison of inferred results with "known" histories. Simulated data would also make it possible to vary the number of families, the number of branches, and the complexity of various features of the model independently, in order to determine how these factors interact.

Systematic investigation of the impact of errors in phylogenetic profiles is another important direction for future investigation [13]. Ancestral reconstructions are sensitive to errors in genome sequencing and assembly, and to thresholding in algorithms used for partitioning domains into families. Genomes that are systematically less well-annotated than others in the same data set may masquerade as genome reduction. To avoid these sources of error as much as possible, we focused on well-studied model organisms, all of which have either finished or high quality draft genomes. Further, because we tested different models on the same data set, the impact on performance comparisons should be limited.

Future simulation studies would be useful in assessing how tree size, tree shape, and taxon sampling influence model performance. Our data set included only six species, with a maximally unbalanced tree topology. We saw the greatest changes in inference on branches near the root of the tree. An important question for future work is whether birth-death models are sensitive to tree shape or to proximity to the root. The model organisms we chose for this study are evolutionarily distant and the branches represent long time intervals. It is likely that better taxon sampling would lead to more accurate reconstructions.

Model choice is of greatest concern when different models lead to fundamentally different outcomes. For our data set, the conclusions implied by the Lineage and Family-Lineage models were fundamentally different for some lineages. This observation could have broad implications. For example, there is mounting evidence for a "revolving door" trend in gene family evolution, characterized by high duplication and loss, but low net change [16]. While we also observed this trend, in our case study, the extent of inferred turnover depends on the model used. For example, the L model inferred heavy traffic through the revolving door in the coelomate lineage, while the FL models inferred none.

Genome streamlining is another trend recently uncovered by birth-death models, in which surges of genome expansion and innovation are followed by widespread genome reduction [25,30]. We see examples of streamlining in our data as well, but only some of these are supported by all models. For example, all four models support a history of genome reduction in Chicken, consistent with similar reports based on other types of evidence [31,32]. In contrast, the Constant and Lineage models inferred substantial losses in the fly lineage, contradicted by both Family-Lineage models, which inferred a net gain in fly. In short, the degree of genome streamlining observed could be influenced by model choice; more complex models may reveal more nuanced patterns of genome expansions and contractions. Our results underscore the importance of revisiting the conclusions of these, and similar, studies using more complex models.

## List of abbreviations used

AIC - Akaike Information Criterion; BIC - Bayesian Information Criterion

## Competing interests

The authors declare that they have no competing interests.

## Authors' contributions

MS and LW designed the statistical study. MS performed all computational analyses. LW provided statistical guidance for the project. MS and DD interpreted the results and wrote the manuscript. All authors read and approved the final manuscript.

## Supplementary Material

Additional file 1 **Original input data**. Tab-delimited table of domain families sizes for all six genomes used in this study: 1471-2164-15-S6-S9-S1.txt Format: TXT Click here for file

Additional file 2 **Supplementary figures and tables**. Additional file containing supplementary figures and tables: 1471-2164-15-S6-S9-S2.pdf Format: PDF Click here for file

## Declarations

Publication charges for this work were funded by NSF grant DBI1262593 to DD.

This article has been published as part of *BMC Genomics *Volume 15 Supplement 6, 2014: Proceedings of the Twelfth Annual Research in Computational Molecular Biology (RECOMB) Satellite Workshop on Comparative Genomics. The full contents of the supplement are available online at http://www.biomedcentral.com/bmcgenomics/supplements/15/S6.
