# Supplementary material for: Robustness of birth-death and gain models for inferring evolutionary events
Source: BMC Genomics. 2014 Oct 17;15(Suppl 6):S9. doi: 10.1186/1471-2164-15-S6-S9 (PMC4239551; doi:10.1186/1471-2164-15-S6-S9)
Supplement: Additional file 2 — Supplementary figures and tables. Additional file containing supplementary figures and tables: 1471-2164-15-S6-S9-S2.pdf Format: PDF [file 1471-2164-15-S6-S9-S2.pdf]

Supplementary Figures and Tables

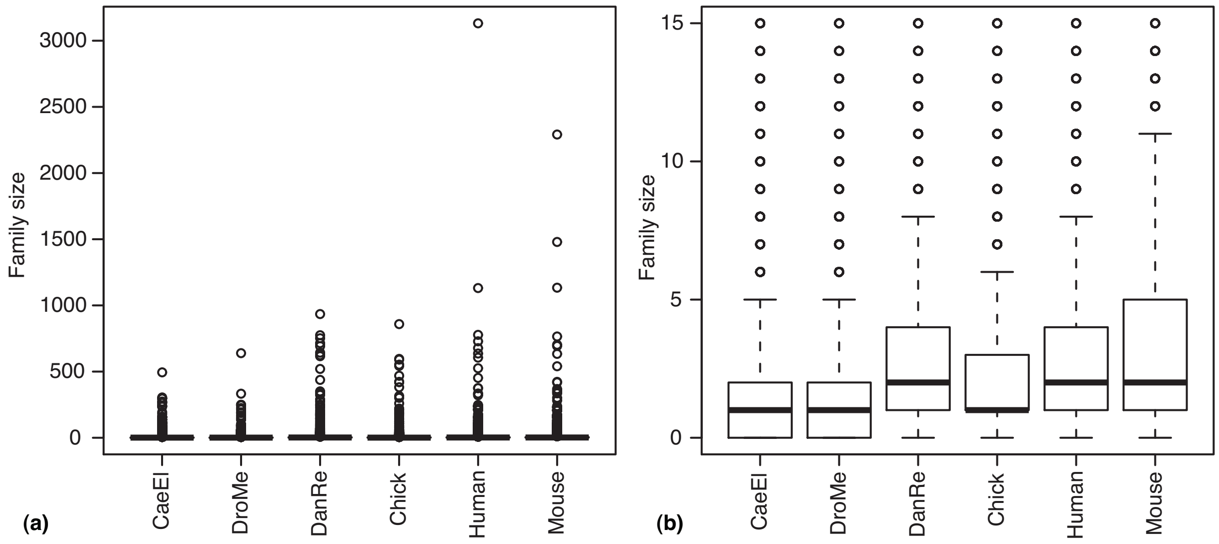

Figure S1 Distribution of family sizes for each contemporary species. (a) Full view and **(b)** a detailed view showing families with 15 or fewer members.

Table S1 Log-likelihood, parameters, and AIC and BIC scores for the original data set. [a] The number of iterations to reach convergence in each stage.

Method	Log-Likelihood	Parameters	AIC	BIC	Iterations[a]
C	-55736.10	4	111480.21	111520.94	21
FO2	-45684.96	12	91393.93	91516.12	21
L	-49115.62	31	98293.23	98608.91	30
FL2	-42542.13	39	85162.27	85559.41	26
FL2242	-42443.18	41	84968.36	85385.87	20
FL3	-42116.51	43	84319.01	84756.88	26
FL4	-42008.55	47	84111.10	84589.70	12
FL5	-41963.22	51	84028.44	84547.77	9

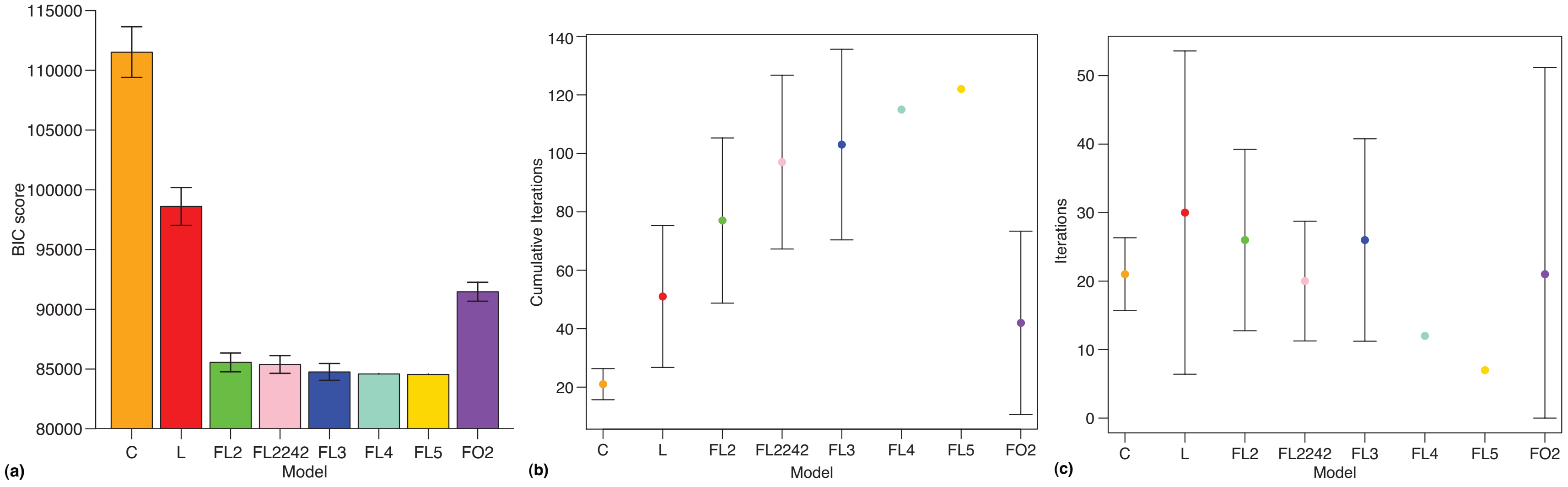

Figure S2 Parameter estimation and convergence (a) BIC scores for the C (orange), L (red), FL2 (green), FL2242 (pink), FL3 (blue), FL4 (teal), FL5 (yellow), and FO2 (purple) models. The number of **(b)** total iterations and **(c)** iterations per stage required to reach convergence for each model stage. Error bars represent standard errors calculated from the bootstrap replicates.

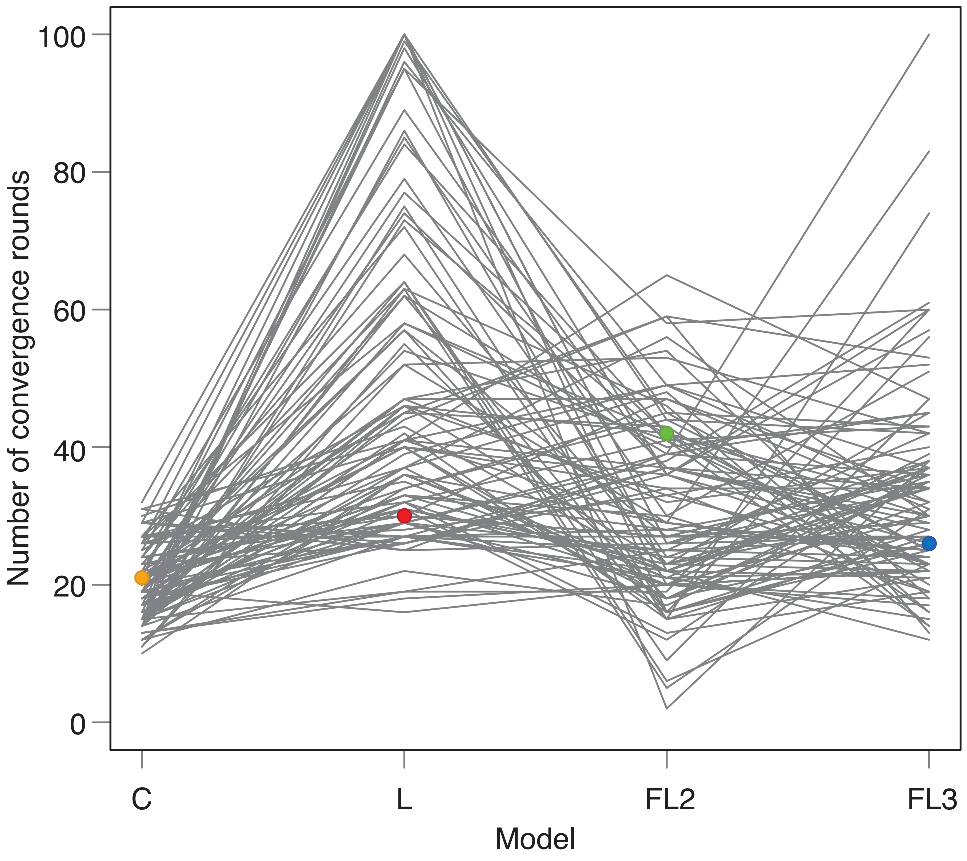

Figure S3 Distribution of iterations over all bootstrap replicates. The number of iterations to reach convergence for each stage (not cumulative). Each line represents one bootstrap replicate. Iterations to convergence for the original data under the C, L, FL2, and FL3 models are show as points in orange, red, green, and blue, respectively.

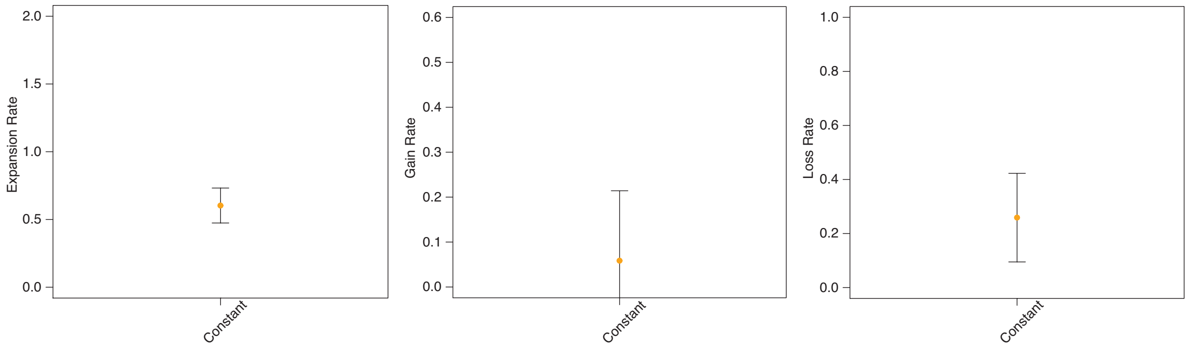

Figure S4 C model event rates Estimated event rates (colored dots) under the C model. Error bars represent standard errors calculated from the bootstrap replicates.

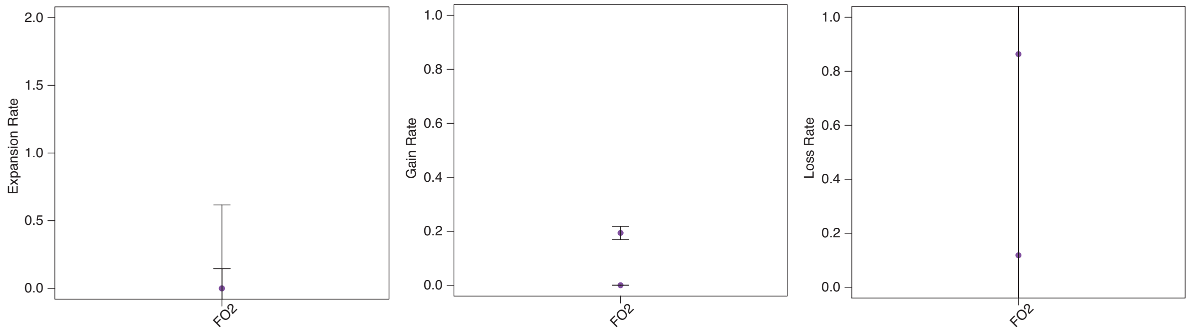

Figure S5 FO2 model event rates Estimated event rates (colored dots) under the FO2 model. Error bars represent standard errors calculated from the bootstrap replicates.

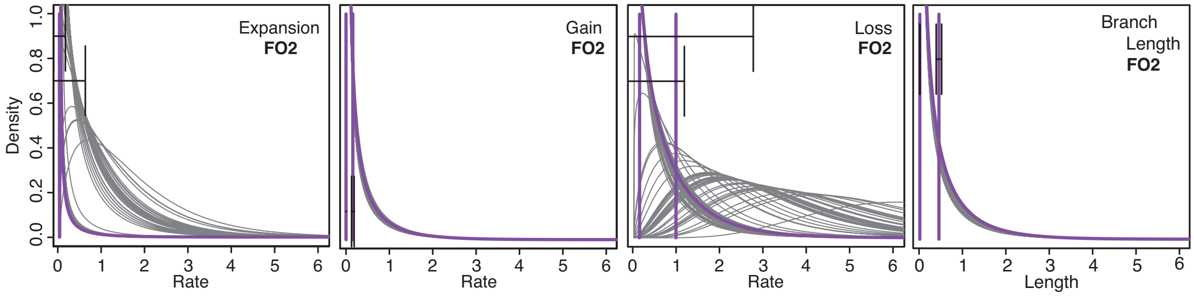

Figure S6 Family-specific parameter distributions under the FO2 model. Estimated distributions for the family components of the Expansion rate, Gain rate, Loss rate, and branch length parameters under the FO2 model. Distribution for the original data shown in purple. Distributions for all bootstrap replicates shown in gray. Purple vertical lines represent the mean value of the estimator for each category, based on the original data, for the FO2 model. Error bars represent standard errors calculated from the bootstrap replicates.

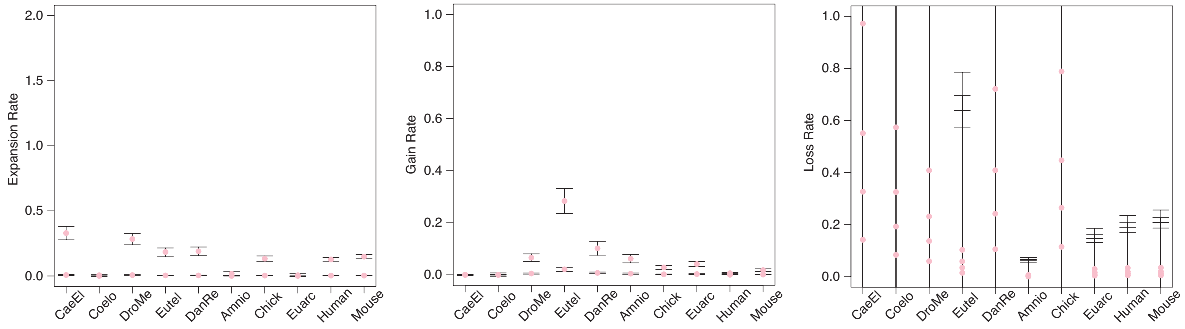

Figure S7 FL2242 model event rates Estimated event rates (colored dots) under the FL2242 model. Error bars represent standard errors calculated from the bootstrap replicates.

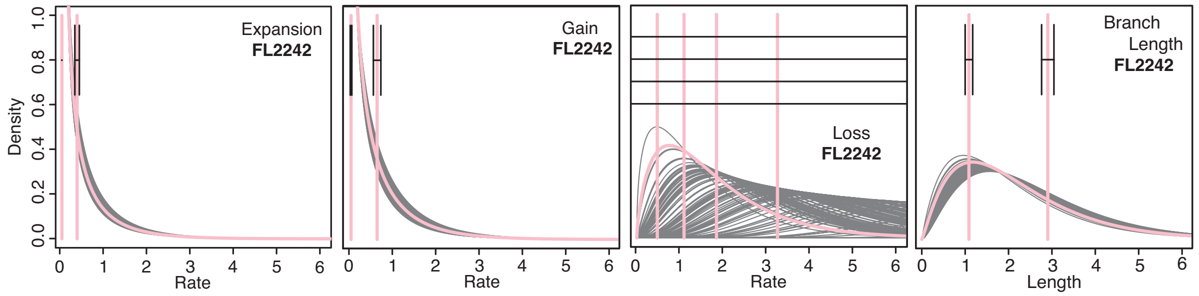

Figure S8 Family-specific parameter distributions under the FL2242 model. Estimated distributions for the family components of the Expansion rate, Gain rate, Loss rate, and branch length parameters under the FL2242 model. Distribution for the original data shown in pink. Distributions for all bootstrap replicates shown in gray. Pink vertical lines represent the mean value of the estimator for each category, based on the original data, for the FL2242 model. Error bars represent standard errors calculated from the bootstrap replicates.

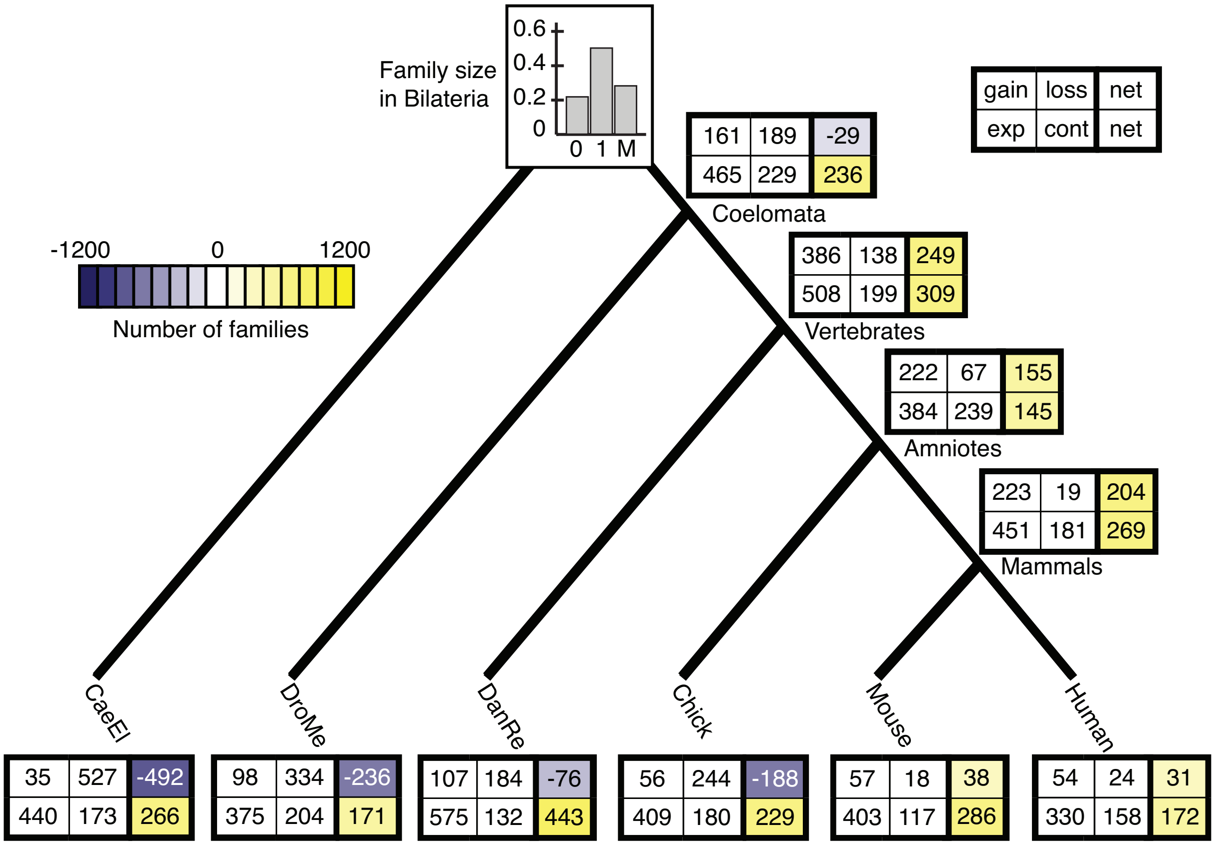

Figure S9 Expected events along each branch under the C model. The expected number of families gained, lost, expanded (exp), and contracted (cont), as well as the net change. The expected fraction of families with 0, 1, or More than one (M) domain in the bilaterian ancestor is shown on the root.

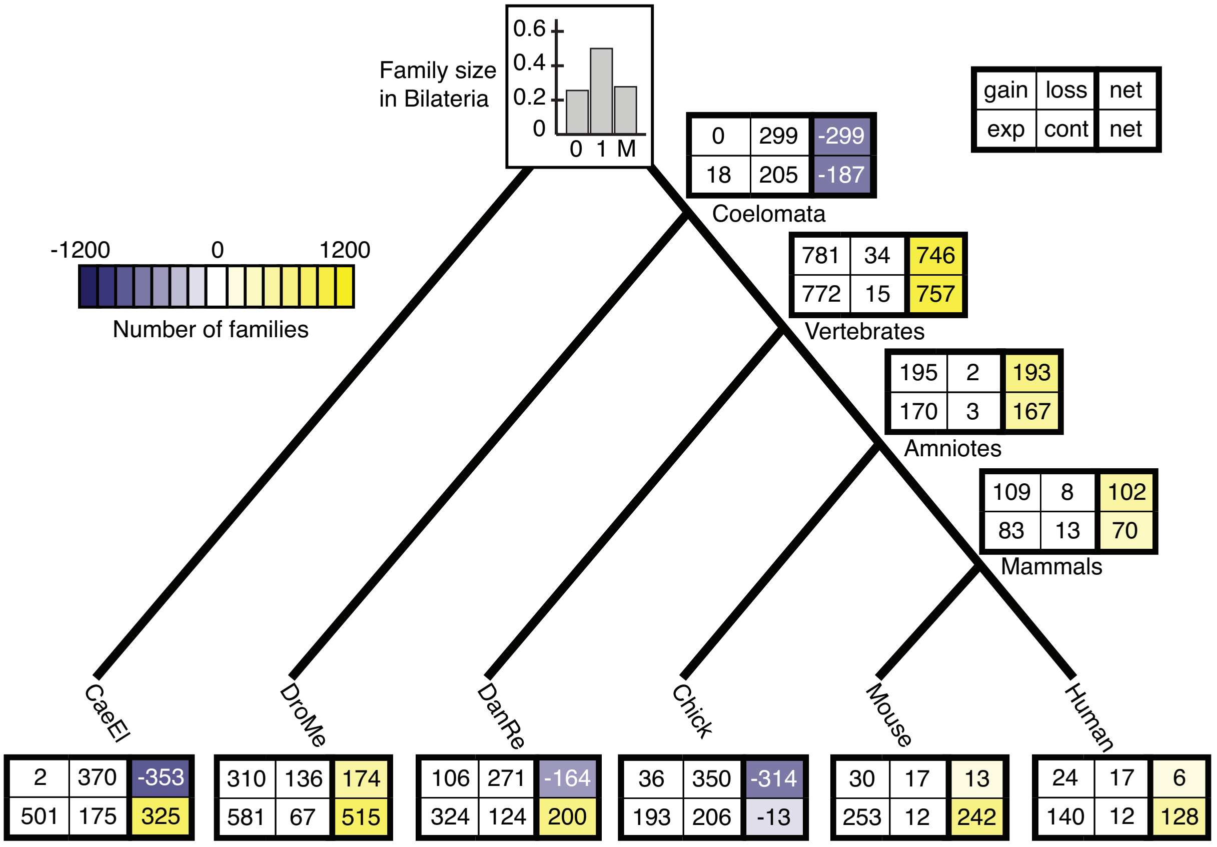

Figure S10 Expected events along each branch under the FL3 model. The expected number of families gained, lost, expanded (exp), and contracted (cont), as well as the net change. The expected fraction of families with 0, 1, or More than one (M) domain in the bilaterian ancestor is shown on the root.

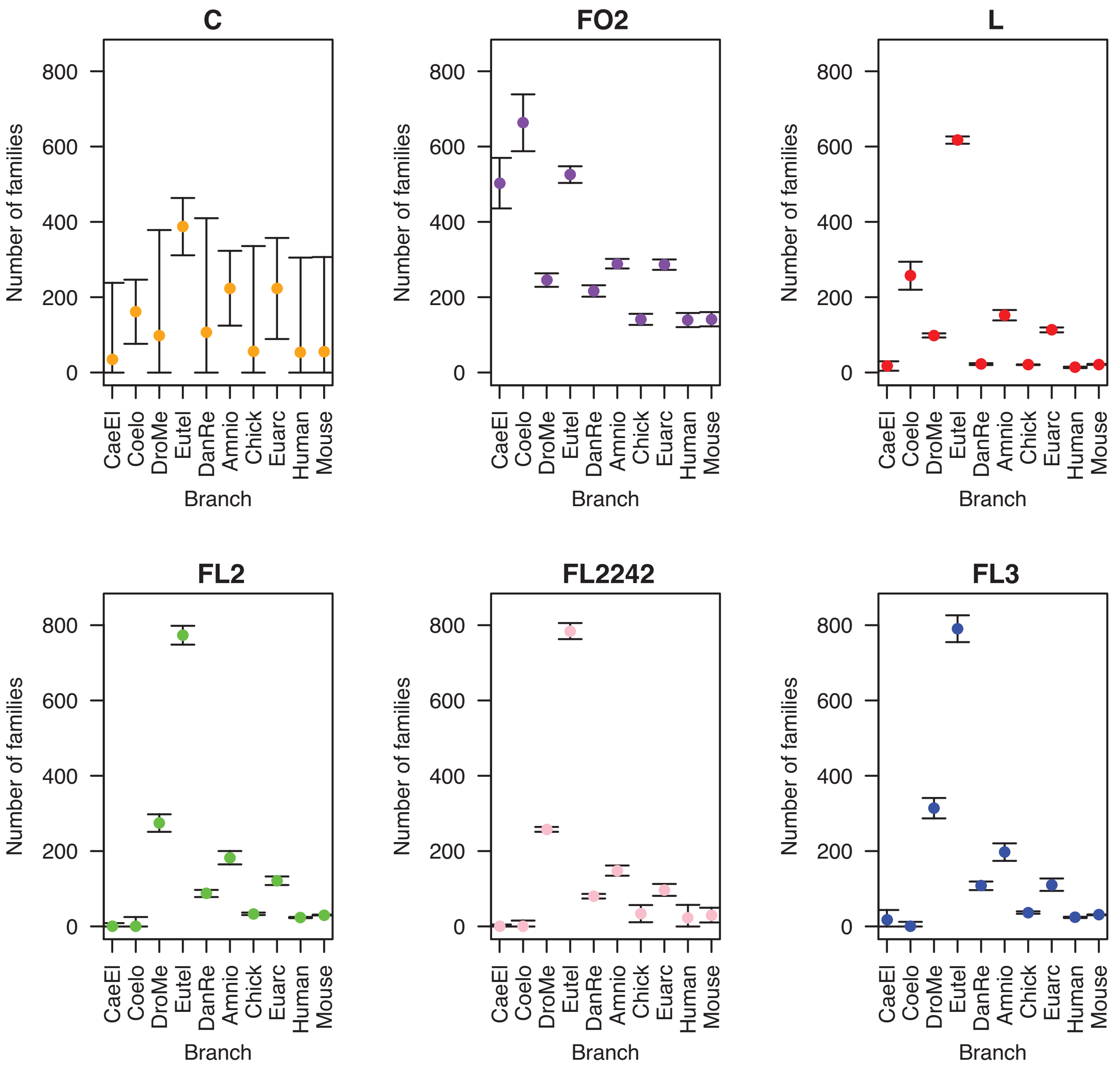

Figure S11 Expected gains. The expected number of families gained along each branch of the species tree under the C, FO2, L, FL2, FL2242, and FL3 models. Error bars represent standard errors calculated from the bootstrap replicates.

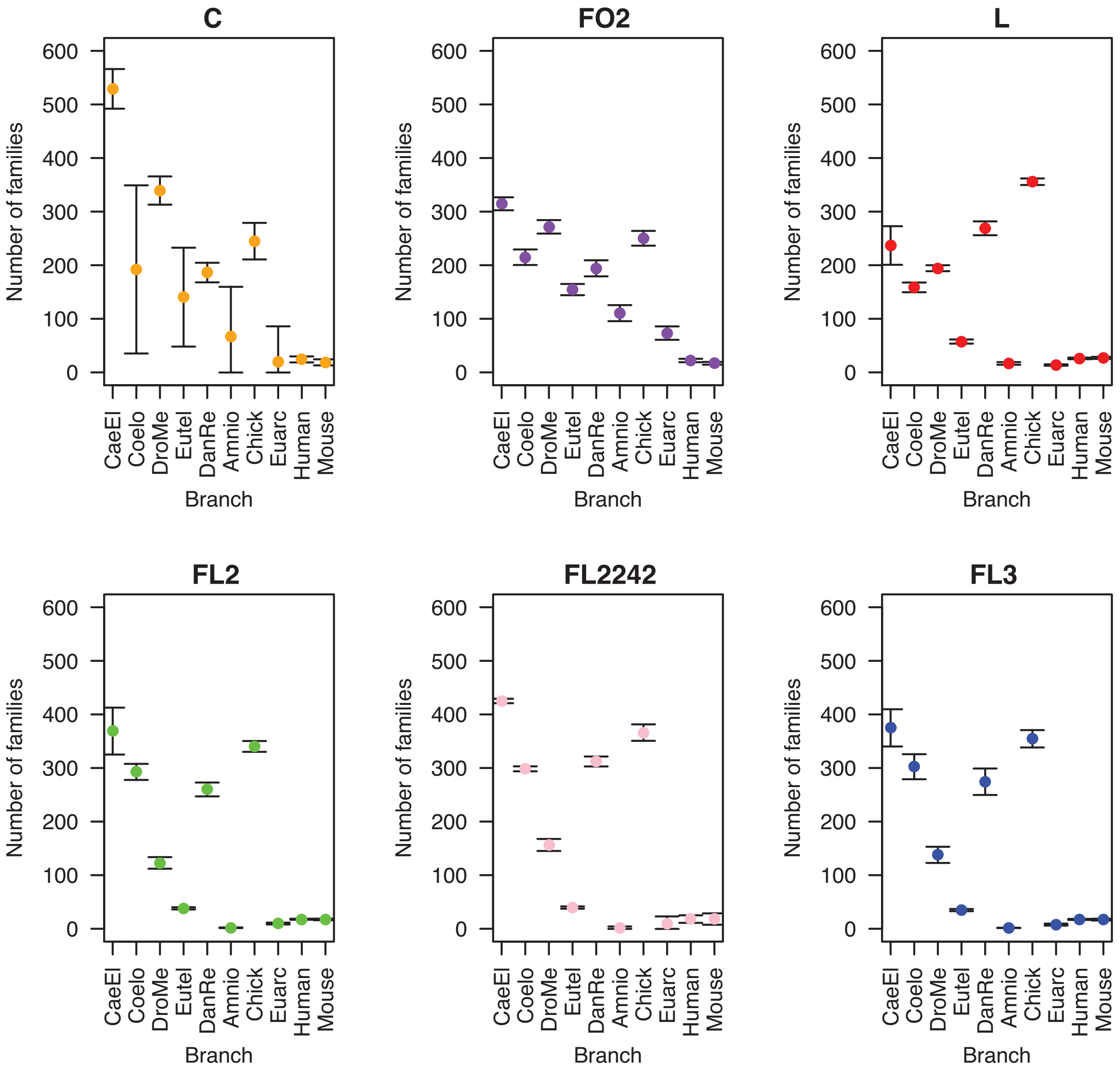

Figure S12 Expected losses. The expected number of families lost along each branch of the species tree under the C, FO2, L, FL2, FL2242, and FL3 models. Error bars represent standard errors calculated from the bootstrap replicates.

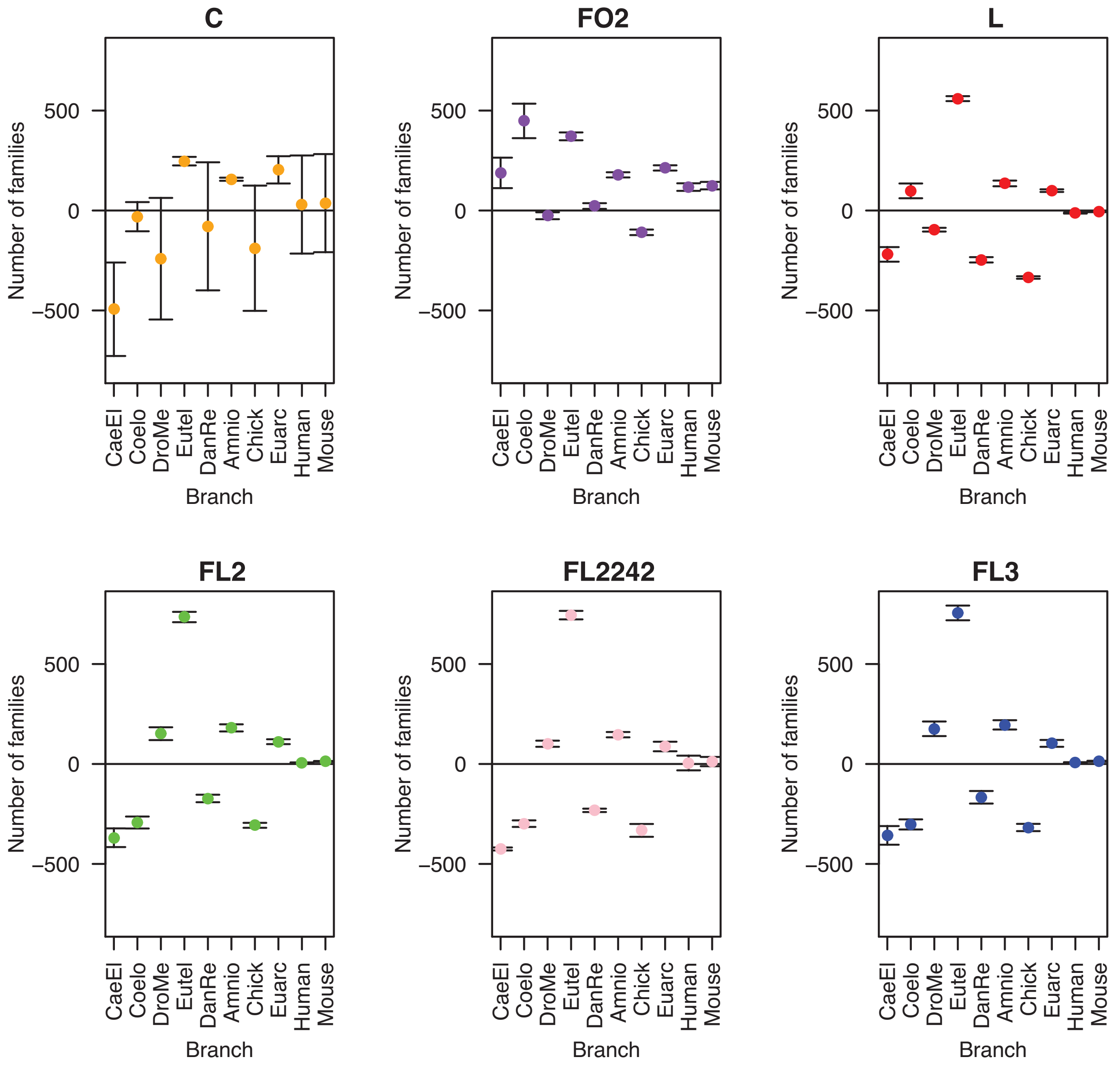

Figure S13 Expected change in family content. Net change in the expected number of families present along each branch of the species tree under the C, FO2, L, FL2, FL2242, and FL3 models. Error bars represent standard errors calculated from the bootstrap replicates.

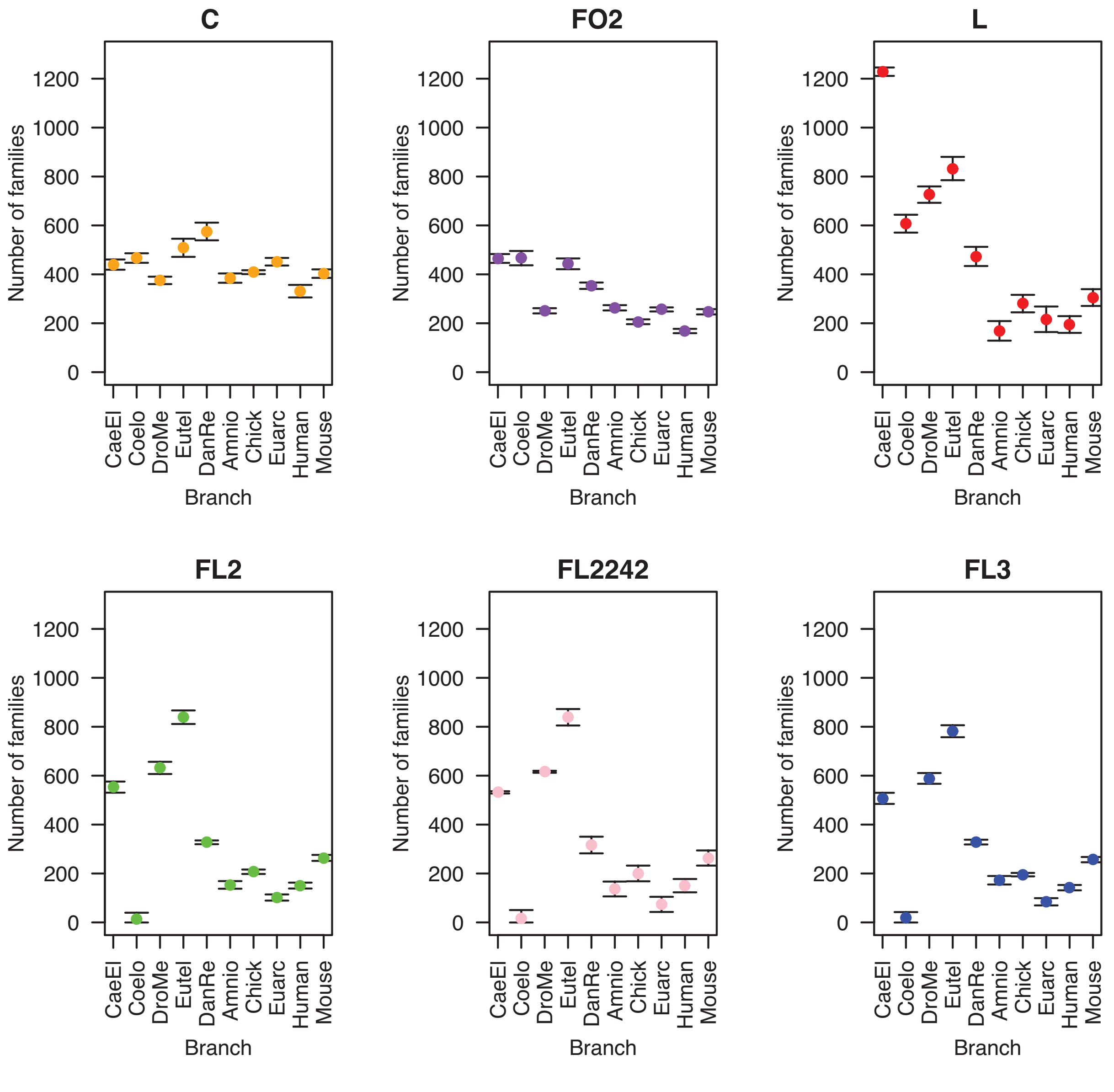

Figure S14 Expected expansions. The expected number of families that expanded along each branch of the species tree for the C, FO2, L, FL2, FL2242, and FL3 models. Error bars represent standard errors calculated from the bootstrap replicates.

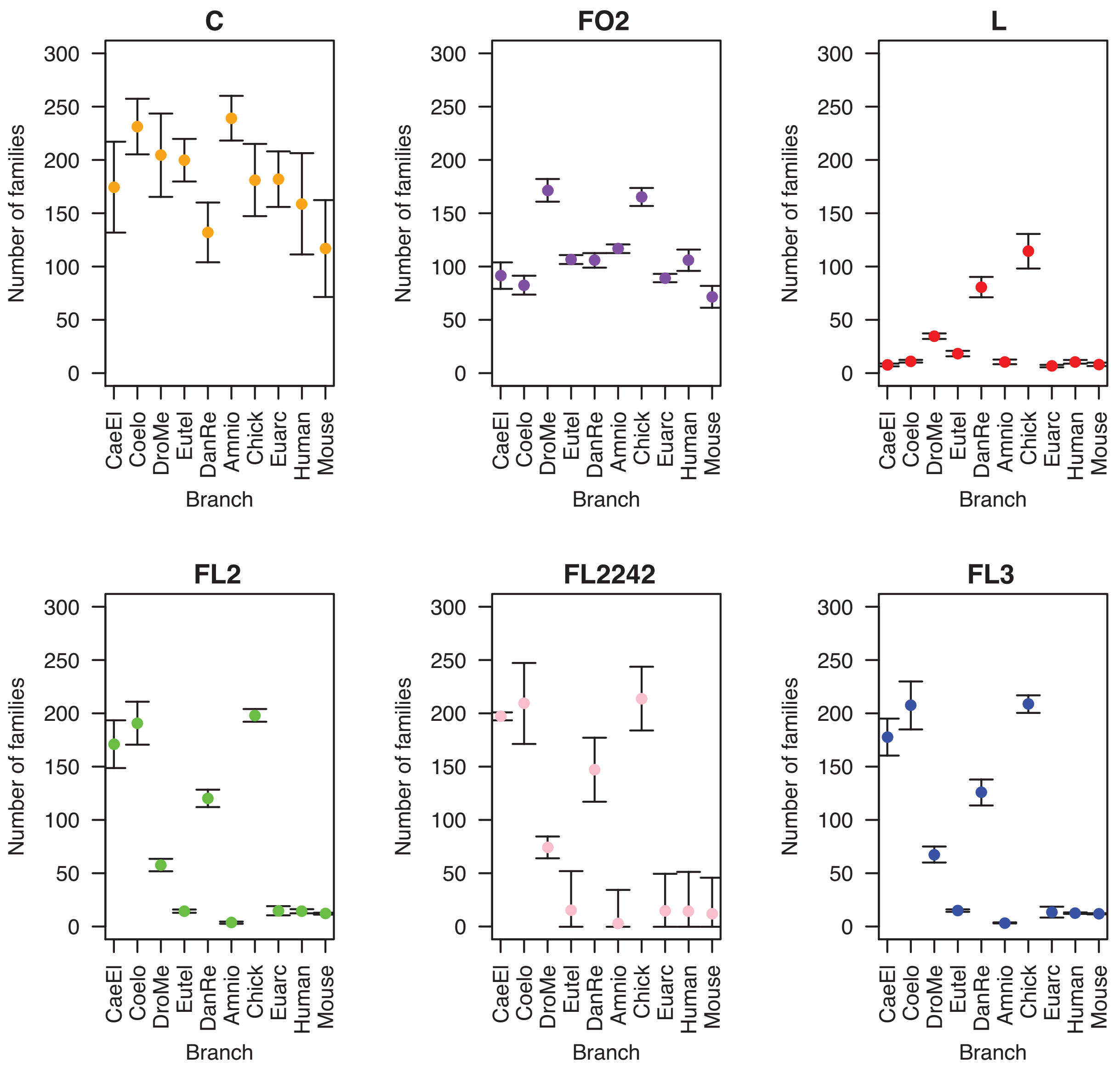

Figure S15 Expected contractions. The expected number of families that contracted along each branch of the species tree under the C, FO2, L, FL2, FL2242, and FL3 models. Error bars represent standard errors calculated from the bootstrap replicates.

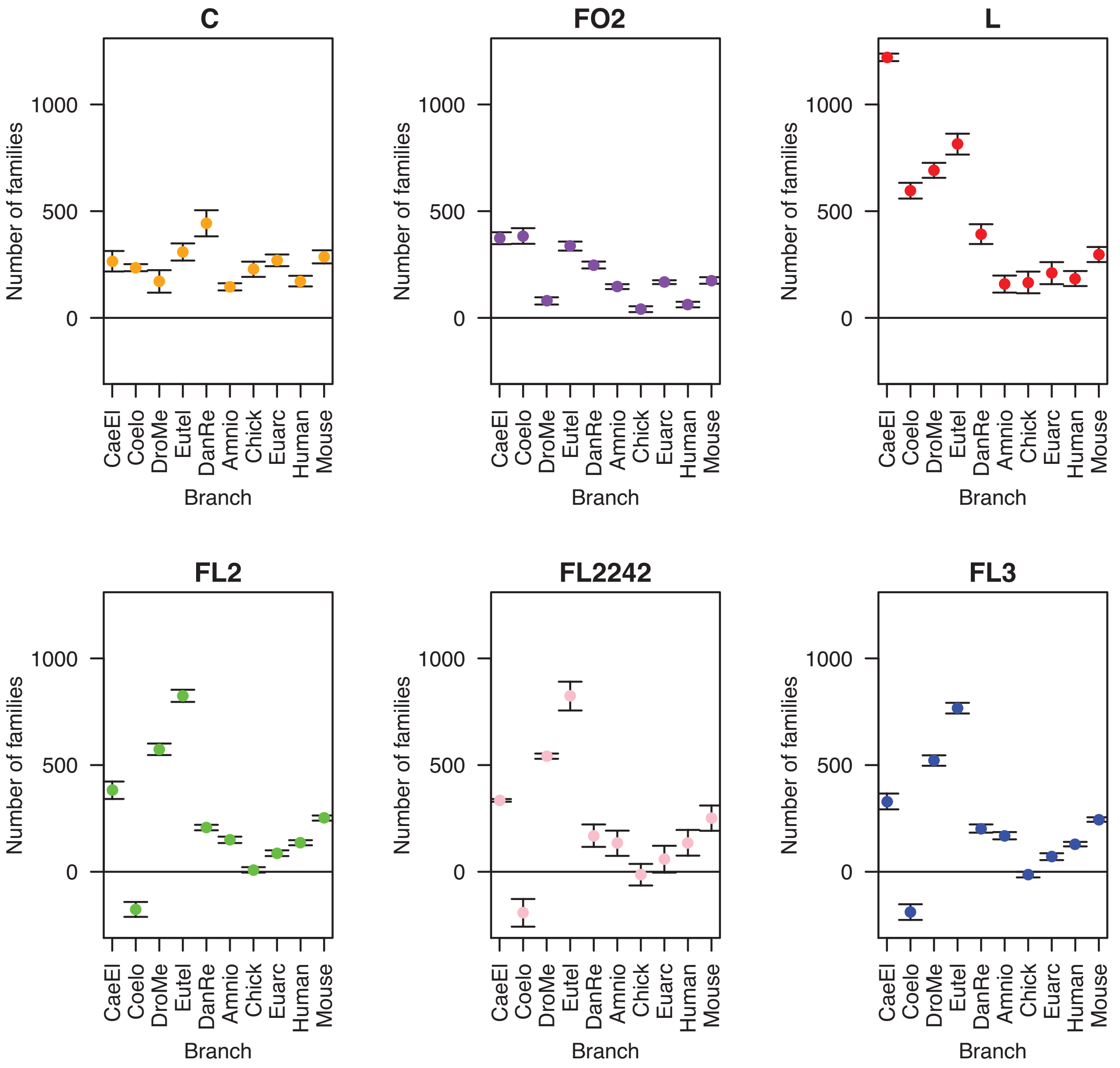

Figure S16 The difference in the expected number of expansions and contractions. The expected number of families that expanded minus the expected number of families that contracted on each branch of the species tree under the C, FO2, L, FL2, FL2242, and FL3 models. Error bars represent standard errors calculated from the bootstrap replicates.
